# Positronium Confined in Nanocavities: The Role of Electron Exchange Correlations

**DOI:** 10.3390/nano11092350

**Published:** 2021-09-10

**Authors:** Fabrizio Castelli, Giovanni Consolati, Giacomo Tanzi Marlotti

**Affiliations:** 1 Department of Physics “Aldo Pontremoli”, Università Degli Studi di Milano, Via Celoria 16, I-20133 Milano, Italy; giacomo.tanzi@unimi.it; 2 INFN, Sezione di Milano, Via Celoria 16, I-20133 Milano, Italy; giovanni.consolati@polimi.it; 3 Department of Aerospace Science and Technology, Politecnico di Milano, Via La Masa 34, I-20156 Milano, Italy

**Keywords:** nanoporous materials, positronium, exchange correlations

## Abstract

Positronium atoms (Ps) are commonly employed as a probe to characterize nanometric or subnanometric voids or vacancies in nonmetallic materials, where Ps can end up confined. The annihilation lifetime of a trapped Ps is strongly modified by pickoff and depends on the cavity size and on the electron density in the confining cavity surface. Here, we develop a theory of the Ps annihilation in nanocavities based on the fundamental role of the exchange correlations between the Ps-electron and the outer electrons, which are not usually considered but must be considered to correctly theorize the pickoff annihilation processes. We obtain an important relation connecting the two relevant annihilation rates (for the *p*-Ps and the *o*-Ps) with the electron density, which has the property of being totally independent of the geometrical characteristics of the nanoporous medium. This general relation can be used to gather information on the electron density and on the average cavity radius of the confining medium, starting from the experimental data on PALS annihilation spectra. Moreover, by analyzing our results, we also highlight that a reliable interpretation of the PALS spectra can only be obtained if the rule of 1/3 between the intensities of *p*-Ps and *o*-Ps lifetimes can be fulfilled.

## 1. Introduction

Positronium atom (Ps), the hydrogen-like bound state of an electron and a positron, was the subject of extensive studies in recent years in the research field of structural analysis of nanoporous materials, with special regards to insulators and molecular solids. In these materials, Ps is usually confined in nanostructured free spaces, defects, or vacancies after formation by implanting positrons. Information about these nanometric and subnanometric porous structures can be derived from the modification of confined Ps annihilation lifetimes, which turn out to be deeply different from the corresponding vacuum values. In particular, Ps lifetimes depend directly on the geometrical and on the physical characteristics of the subnanometric voids or cavities, i.e., their average size and the electron density in surface proximity, respectively. For the measurement of Ps lifetimes, the technique of positron annihilation lifetimes spectroscopy (PALS) [1] is the most commonly used between the few methods available to obtain this information.

As a matter of fact, depending on Ps internal spin configuration, in a vacuum we have two distinct annihilation times: a short component $\tau_{2\gamma }=\lambda_{2\gamma }^{-1}=0.125\;\mathrm{ns}$ and a long component $\tau_{3\gamma }=\lambda_{3\gamma }^{-1}=142\;\mathrm{ns}$ for the singlet (*para*-Ps, or *p*-Ps) and triplet (*ortho*-Ps, or *o*-Ps) state, respectively, where $\lambda_{2\gamma }$ and $\lambda_{3\gamma }$ are the corresponding annihilation rates [2]. The subscripts 2γ and 3γ indicate annihilation with the production of 2 or 3 gamma rays, respectively. When Ps is confined in porous materials, both short and long lifetime components in PALS spectra are modified, reflecting the presence of a statistical mixture of Ps states modified both in spin configuration and spatial wavefunction.

The theoretical representation of annihilation rates of Ps inside nanoporous matter is usually based on approximate one- or two-body descriptions of the so-called *pickoff* process; i.e., the additional possibility of the positron annihilating with an electron in the surroundings. The most used one-body models describing Ps confined in small cavities are based on the Tao–Eldrup (TE) approach [3,4], relating pickoff annihilation rates $\lambda_{\mathrm{po}}$ to cavity sizes by considering Ps as a single quantum particle trapped inside an infinite potential well. These models were extended to describe various cavity geometries and temperature effects [5,6]. On the other hand, two-body models are developed to describe the internal structure of confined Ps by considering separate degrees of freedom for the positron and the electron [7,8,9]. Moreover, fully ab initio treatments of the confined two-particle bound system can in principle be done [10], but they are usually avoided given the huge computational efforts required.

At the basis of every one-body and two-body model is the implicit assumption that Ps interaction with surrounding electrons can be described as a small perturbation, depending only on their density. The external electronic environment only accounts for pickoff annihilations without any correlation with the Ps spatial waveform and spin configuration. But, only the two-body approach is a simple way of theoretically describing the lowering of the contact density (i.e., the electron density at the positron position, governing the annihilation processes) observed for Ps in nanomaterials [11,12]. This feature is usually described by introducing a relative *contact density parameter* $k_{r}$, defined as the probability of finding the Ps–electron at the positron position in units of the vacuum value $k_{0}=1/8\pi a_{0}^{3}$ ($a_{0}$ being the Bohr radius) [11], and is associated with some deformation of the Ps wavefunction in the nanovoids [8,13].

However, the validity of these theoretical treatments in which Ps is considered as a separate “entity”, and the Ps-electron is somehow privileged with respect to external electrons, must be questioned against the requirement of full electron indistinguishability, which has a direct relation to the pickoff annihilation. In fact, complete electron indistinguishability is evident in some materials or compounds exhibiting a single lifetime component (a simple example of a Ps-like system having only one lifetime component is ${\mathrm{Ps}}^{-}$).

In Ref. [14], Tanzi et al. analyze in detail this problem and provide a theoretical framework in which electron indistinguishability can be introduced perturbatively in a natural way, while also preserving the concept of para/ortho Ps atoms essential for the connection with the experimental results on nanoporous materials. Their treatment takes into account explicit spin configurations and positron–electron correlations in the modeling of the Ps-environment system by means of an especially developed symmetry-adapted perturbation theory (SAPT) [15] and using a local density approximation (LDA) to describe the properties of the electron system. Formal expression for annihilation rates are derived, and numerical results on Ps lifetimes and contact density as a function of the small cavity radius and of the surrounding electron density are obtained and interpreted physically.

Here, we resume these formal expressions for Ps pickoff and total annihilation rates, and in the spirit of the TE modeling and by applying suitable approximations, we obtain simple and practical expressions connecting annihilation rates with electron densities. In particular, a fundamental formula directly connecting a proper combination of measurable annihilation rates with the electron density near the cavity surface is derived. This result is completely independent of the geometrical characteristics of the confining cavity and of the form of the confining potential. Therefore, it is of invaluable utility for experimentalists. Application of our theoretical results to PALS experimental data for some solid polymers and molecular crystals is also considered and discussed. By resorting to the spherical approximation for the nanocavities, which is at the hearth of the TE proposal, we obtain some useful hints on the sizes and the radius of the confining free spaces.

Finally, we note that, by analyzing the results of our theory in comparison with that of PALS experimental data, a reliable interpretation of the components of the PALS annihilation spectra can be obtained only if the ratio between the intensities of the short lifetime component (attributed to *p*-Ps) to the long lifetime component (attributed to *o*-Ps) can be imposed as equal to 1/3 during the analysis of the spectra themselves, as expected by the theories on the formation of Ps in vacancies.

## 2. Ps Annihilation Rates and Exchange Correlations

In past literature, the most common expressions describing the measurable annihilation rates of the two possible Ps states confined in nanocavities or in porous matter, denoted $\lambda_{t}$ and $\lambda_{s}$ for *o*-Ps and *p*-Ps, respectively, are constructed by considering on the same foot intrinsic and pickoff contributes [16]:

(1a) $$\begin{array}{cc} \lambda_{t}\; & =\;k_{r}\;\lambda_{3\gamma }+\lambda_{\mathrm{po}}\;, \end{array}$$

(1b) $$\begin{array}{cc} \lambda_{s}\; & =\;k_{r}\;\lambda_{2\gamma }+\lambda_{\mathrm{po}}\;. \end{array}$$

The first term is characterized by $k_{r}$, the relative contact density parameter usually assumed an intrinsic property of the confined Ps. The pickoff contribution $\lambda_{\mathrm{po}}$, identical in both equations, is naturally a *surface* process, and in every model, it is assumed to depend on a geometrical probability, commonly denoted by $P_{\mathrm{out}}$, of finding Ps outside the free-space (*inner*) region defining the nanocavity:

(2) $$\lambda_{\mathrm{po}}=P_{\mathrm{out}}\bar{\lambda }\;,$$

where $\bar{\lambda }$ is a suitable bulk annihilation rate, that can be taken as the weighted average of singlet and triplet decay rates, following the original idea of the TE one-particle model [3,4]:

(3) $$\bar{\lambda }\;=\;\frac{1}{4}\lambda_{2\gamma }+\frac{3}{4}\lambda_{3\gamma }\;=\;2.01\;\;\;{\mathrm{ns}}^{-1}\;.$$

Within this approximation, $\bar{\lambda }$ is independent of the electronic properties of the surrounding medium, which eventually are contained in the expression of $P_{\mathrm{out}}$, and the geometrical parameters of the model are chosen to fit the correct pickoff annihilation rate in real systems.

In some one-particle models, the intrinsic Ps annihilation rate is assumed independent of confinement by simply taking $k_{r}=1$ [17,18,19]. In more refined models [6,20,21]), different prescriptions about the proper way of treating Ps in the *inner* and *surface* regions are introduced. For example, in ref. [20] Ps annihilates with its intrinsic vacuum annihilation rate only in the *inner* part of the cavity, whereas the *surface* region annihilation is dominated by pickoff. In this way, one writes $k_{r}=1-P_{\mathrm{out}}$, i.e., the probability of finding Ps in the *inner* spatial region.

Better characterization of Ps annihilation rates is obtained in two-particle models [8,13,22]. Here, pickoff annihilation is proportional to the probability $P_{\mathrm{out}}^{+}$ of having only the *positron* wandering outside the cavity, which is similar but different from $P_{\mathrm{out}}$. Moreover, it also depends on the electron density in the region around the cavity, which can be different with respect to the average electron density in the bulk. On the other side, intrinsic annihilation is assumed to take place only in the spatial region allowed to the motion of the *electron* bounded to the positron, which can be either extended to the whole space [22] or strictly limited to the *inner* cavity under the effect of strong repulsive Pauli exchange forces with bulk electrons [8,23]. In this way, $k_{r}$ can be associated with possible modifications of the internal spatial structure of Ps wavefunction, and its lowering below the vacuum value usually found in experiments can be justified. Moreover, from these models, information on nanopore sizes, positron work functions, and electron density near the cavity surface can be obtained from experimental PALS spectra.

On the other hand, all these models do not depend on the effective spin configuration of the Ps-electron, which therefore does not affect the pickoff annihilation behavior of the Ps-positron in the outer region, and as a result, *o*-Ps and *p*-Ps have the same pickoff annihilation rate. Hence, pickoff processes are exclusively represented by the term $\lambda_{\mathrm{po}}$ in both Equation (1), while $k_{r}$ is the only parameter associated to possible modifications of the internal spatial structure of Ps wavefunction. Another way to discuss this point is to observe that no exchange correlation effects, due to the Pauli exclusion principle, are ascribed to the Ps-electron in relation to the outer electrons. As a consequence, the positron can annihilate with the same probability with all surrounding electrons independently from their spin, as stated in the Expression (3). On the contrary, if exchange correlation effects are considered, the positron annihilates, with different probability, with electrons having opposite or parallel spin with respect to Ps-electron, with the consequence of different pickoff annihilation rates for *o*-Ps and *p*-Ps.

To our knowledge, the possibility of having different pickoff annihilation rates was only noted by Mogensen and Eldrup in 1977 [24], but never further investigated. Anyway, this represents a minor problem to the positronium community since the presence of an exchange effect has a negligible influence on the total annihilation rate of the *o*-Ps system, i.e., the easily measurable long-life component of PALS spectra, because usually $\lambda_{\mathrm{po}}\gg \lambda_{3\gamma }$. On the contrary, for the short-life component belonging to the *p*-Ps system, pickoff and intrinsic annihilation rates may be comparable.

A clarification of the role of exchange correlation effects on differentiating *o*-Ps and *p*-Ps pickoff annihilation rates can be answered theoretically with the techniques of many-body quantum mechanics. Recently, Tanzi et al. [14] used a rigorous SAPT (symmetry–adapted perturbation theory) framework [15] to deal with the antisymmetrization of the wave function of the external system of N electrons. A summary of the method and the resulting expressions is presented in Appendix A. In [14], they show that, up to first order in exchange contributions, the formal expressions for the total annihilation rates of *o*-Ps and *p*-Ps interacting with the external electron system can be written as:

(4a) $$\begin{array}{cc} \lambda_{t} & \;=\;\lambda_{3\gamma }+\frac{\lambda_{\mathrm{ex}}^{3\gamma }}{1-S}+\frac{\lambda_{\mathrm{sym}}}{1-S}\;, \end{array}$$

(4b) $$\begin{array}{cc} \lambda_{s} & \;=\;\lambda_{2\gamma }+\frac{\lambda_{\mathrm{ex}}^{2\gamma }}{1-S}+\frac{\lambda_{\mathrm{sym}}}{1-S}\;, \end{array}$$

where the normalization term *S* is a wavefunction overlap factor, whose explicit expression is given in the Appendix A. Equation (4) shows that all the pickoff annihilation contributions of a Ps atom interacting with an environment system can be grouped into two distinct terms. The term $\lambda_{\mathrm{sym}}$ represents the contribution to the annihilation rate that can be directly linked to the presence of external electrons. This contribution is symmetric with respect to Ps spin configuration, i.e., it is the same for *o*-Ps and *p*-Ps and, as we will show in the next Section, it is very similar to the “standard” pickoff annihilation rate of Equation (2). The other terms $\lambda_{\mathrm{ex}}^{3\gamma }$ and $\lambda_{\mathrm{ex}}^{2\gamma }$ in Equation (4) derive from exchange effects between the Ps internal electron and the surrounding ones. As such, their contribution to annihilation rate strongly depends on the symmetry of the Ps-environment wavefunction, hence, giving different results for different Ps spin configurations.

We just note that these expressions can be considered of the same family of the general Equation (1) as long as one realizes that these last can be written as:

(5a) $$\begin{array}{cc} \lambda_{t} & =\lambda_{3\gamma }+\left[\left(k_{r}-1\right)\lambda_{3\gamma }+\lambda_{\mathrm{po}}\right]\;, \end{array}$$

(5b) $$\begin{array}{cc} \lambda_{s} & =\lambda_{2\gamma }+\left[\left(k_{r}-1\right)\lambda_{2\gamma }+\lambda_{\mathrm{po}}\right]\;, \end{array}$$

where the term in square brackets is the overall contribution to the annihilation due to the external electrons (the total pickoff), and each term of the sum can be put in direct correspondence with the terms of Equation (4). This observation makes also clear that the accurate Ps annihilation theory derived in [14] can give formal expressions for the relative contact density $k_{r}$ and for the *geometrical* pickoff contribute $\lambda_{\mathrm{po}}$ of Equation (2).

## 3. The Effective Electron Density Felt by a Confined Ps

It is of paramount importance to put the formal Expressions (4) in a form that can be directly related to PALS experimental measurements. Indeed, with a little algebra, the overlap factor disappears, and these Equation are transformed into this single relation:

(6) $$\frac{\lambda_{\mathrm{ex}}^{2\gamma }+\lambda_{\mathrm{sym}}}{\lambda_{\mathrm{ex}}^{3\gamma }+\lambda_{\mathrm{sym}}}\;=\;\frac{\lambda_{s}-\lambda_{2\gamma }}{\lambda_{t}-\lambda_{3\gamma }}\;\equiv \;K_{M}\left(\lambda_{s},\lambda_{t}\right)\;,$$

where the quantity $K_{M}\left(\lambda_{s},\lambda_{t}\right)$ defined on the right-side of the relation contains only measurable or known quantities, while the ratio on the left-side contains only the exchange contributions, which in turn depend on the electronic density n(r), the one-body reduced density matrix (1RDM) $\Gamma^{\left(1\right)}\left(x;y\right)$ (see Appendix A), and geometrical overlap factors. Hence, this relation allows to directly connect theoretical calculations with experimental data.

To make effective these considerations and to derive reasonable and practical expressions for the involved quantities, it is necessary to introduce some further approximations. In fact, the only assumption we made about the system interacting with Ps is that of uniform spin distribution (Equation (A11)) of external electrons, a condition that translates in the absence of local spin polarization around the cavity region in the unperturbed ground state of the system. On the other hand, no assumption on the form of the electrons wavefunction ϕ was done so that the formulation of the annihilation rates as given in Equation (4) is completely general.

To go on, we use the same concepts discussed in Section 2, i.e., a vacuum-like separable Ps-system whose wavefunction exhibits a probability $P_{\mathrm{out}}$ of finding itself into a certain small *interaction region* Ω around the confining nanocavity. This interaction region is considered filled with a near homogeneous electron gas. Given that this is a low-order approximation, the relative motion part of the two Ps particles is taken as in vacuum, while the center of mass is only responsible for defining $P_{\mathrm{out}}$. For the current results, the specific form of Ps wavefunction is not important as long as we assume the usual factorization:

(7) $$\Psi \left(r_{p},r_{e}\right)\;=\;\psi \left(r_{pe}\right)\Psi \left(R_{pe}\right)\;,$$

where $\psi (r_{pe})$ is the relative wavefunction, $\Psi (R_{pe})$ the center of mass wavefunction, and we use, here and in the following, the compact notation $r_{pe}=r_{p}-r_{e}$ for the relative coordinate, and $R_{pe}=\left(r_{p}+r_{e}\right)/2$ for the center of mass coordinate.

We neglect all Coulomb potentials, except the one leading to the bound Ps atom, because these potentials describe an overall effect of interaction producing the confinement of Ps; hence, determining the center of mass wavefunction $\Psi (R_{pe})$. However, the radial part of the relative wavefunction can be taken as those of the unperturbed Ps (a 1S orbital):

(8) $$\psi \left(r_{pe}\right)=\sqrt{k_{0}}\;\;e^{-\frac{r_{pe}}{2a_{0}}}\;,$$

where $k_{0}=1/8\pi a_{0}^{3}$, the vacuum contact density ($r_{pe}=\left|r_{pe}\right|$).

On the other side, giving accurate expressions for the electron density function n(r) interacting with Ps in the region Ω, and for the relative 1RDM, is an extremely complicated task if one has to consider all the interactions naturally present in the system. For example, the positron–electron attraction would lead to an enhancement of the electron density at the positron position [25], therefore increasing the annihilation rate. Without any knowledge of the amount of this enhancement, we can just define a quantity $\rho_{e}$ to be the *effective electron density felt* by the Ps in the interacting region, and treat it as a free parameter to be determined later, as we will see that it is strongly connected to experimental data. To determine the 1RDM, instead, we consider the local density approximation (LDA) as detailed in the Appendix B (see Equation (A16)), which gives an explicit formula for this quantity depending on the Fermi momentum $k_{F}={\left(3\pi^{2}\rho_{e}\right)}^{1/3}$, appropriate to the effective electron density, and on the function $B(k_{F}\;r_{pe})$ there defined.

With these statements, we obtain approximate explicit forms for the relevant quantities. Let’s introduce the quantity $P_{\mathrm{out}}$, defined as the probability of finding the Ps center of mass in the limited interaction region outside the confining cavity,

(9) $$P_{\mathrm{out}}\;\equiv \;\int_{\Omega }\;{\left|\Psi \left(R\right)\right|}^{2}\;\;d^{3}R\;.$$

The symmetric contribution to the annihilation in Equation (A14) becomes, assuming $n\left(r\right)≃\rho_{e}$ uniformly in the small region Ω:

(10) $$\begin{array}{cc} \lambda_{\mathrm{sym}}\;= & \;8\pi a_{0}^{3}\;\;\bar{\lambda }\;\int {\left|\Psi \left(r_{p},r_{e}\right)\right|}^{2}n\left(r_{p}\right)\;d^{3}r_{p}\;d^{3}r_{e}\\ \;\approx & \;\bar{\lambda }\;\frac{\rho_{e}}{k_{0}}\;P_{\mathrm{out}}\;\int \;{\left|\psi \left(r_{pe}\right)\right|}^{2}\;\;d^{3}r_{pe}\;=\;\bar{\lambda }\;\frac{\rho_{e}}{k_{0}}\;P_{\mathrm{out}}\;. \end{array}$$

The exchange contribution for *o*-Ps in Equation (A15) becomes:

(11) $$\begin{array}{cc} \lambda_{\mathrm{ex}}^{3\gamma }\; & =\;-8\pi a_{0}^{3}\;\lambda_{3\gamma }\int \;\Psi^{*}\left(r_{p},r_{e}\right)\Psi \left(r_{p},r_{p}\right)\;\Gamma_{↑↑}^{\left(1\right)}\left(r_{e};r_{p}\right)\;\;d^{3}r_{p}\;d^{3}r_{e}\\ \; & \;\approx -\lambda_{3\gamma }\;\frac{\rho_{e}}{2\sqrt{k_{0}}}\;P_{\mathrm{out}}\int \;\psi \left(r_{pe}\right)B\left(k_{F}\;r_{pe}\right)\;\;d^{3}r_{pe}\;, \end{array}$$

while for *p*-Ps the exchange contribution is simply obtained by interchanging $\lambda_{\mathrm{ex}}^{3\gamma }\;↔\;\lambda_{\mathrm{ex}}^{2\gamma }$. Finally, the overlap parameter from Equation (A13) becomes:

(12) $$S\;\approx \frac{\rho_{e}}{2}\;P_{\mathrm{out}}\;\int \;\psi \left(r_{pe}\right)\psi \left(r_{p1}\right)B\left(k_{F}\;r_{e1}\right)\;\;d^{3}r_{pe}\;d^{3}r_{p1}\;.$$

In this picture, these formulas represent the first-order interaction of the Ps with the uniform gas of electrons in which it is immersed, with the consequence of a modification of the annihilation behavior. The interaction probability is characterized by the geometrical factor $P_{\mathrm{out}}$, which is the probability that the two systems enter in contact. Moreover, both the symmetric and the exchange annihilation contributions, and also the overlap factor, all result in being proportional to $P_{\mathrm{out}}$. On the other hand, the proper electron density $\rho_{e}$ felt by the Ps is present directly as a proportionality factor in all these quantities, but it is also present in the definition of the Fermi momentum $k_{F}$.

An interesting observation can be immediately extracted: while the symmetric contribution to pickoff annihilation is naturally a positive quantity, independent of spin configuration, the exchange contributions $\lambda_{\mathrm{ex}}^{3\gamma }$ and $\lambda_{\mathrm{ex}}^{2\gamma }$ are negative quantities, tending to lower the total annihilation rates, and hence, showing something like a *shielding effect* on the Ps. This effect can be interpreted as a consequence of the repulsive Pauli exchange forces acting on the electron system. The positron will most likely annihilate with electrons having opposite spin with respect to Ps–electron, with the consequence of different pickoff annihilation rates for *o*-Ps and *p*-Ps due to spin exchange. This can be easily understood because the function $B(k_{F}\;r_{pe})$ is essentially positive in the relevant integration range.

A second fundamental consequence of the above approximate expressions is that the left-hand side of the relation in Equation (6) can be rewritten without direct dependence on $P_{\mathrm{out}}$, as shown below. Note that a similar observation cannot be valid for $\rho_{e}$ because its presence in $k_{F}$.

**Figure 1:**
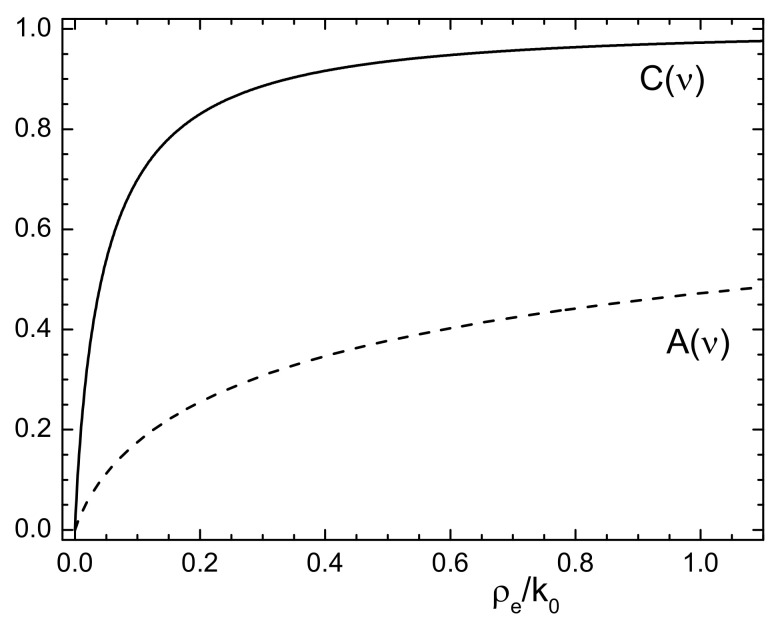
Plot of the functions A(ν) and C(ν) of Equation (13), where $\nu ={\left(3\pi \rho_{e}/k_{0}\right)}^{1/3}$, as a function of the normalized electron density $\rho_{e}/k_{0}$.

Let’s formalize in a suitable way these statements. Firstly, we define two adimensional auxiliary functions summarizing the dependence of the rates $\lambda_{\mathrm{ex}}^{3\gamma },\lambda_{\mathrm{ex}}^{2\gamma }$ and of the overlap factor *S* on the electron density normalized with respect to the vacuum contact density $\rho_{e}/k_{0}$:

(13a) $$\begin{array}{cc} A(\nu )\; & =\;\frac{\rho_{e}}{2\sqrt{k_{0}}}\;\int \;\psi \left(r_{pe}\right)B\left(k_{F}\;r_{pe}\right)\;\;d^{3}r_{pe}\;=\;\frac{2}{\pi }\left[\arctan\left(\nu \right)-\frac{\nu }{1+\nu^{2}}\right]\;, \end{array}$$

(13b) $$\begin{array}{cc} C(\nu )\; & =\;\frac{\rho_{e}}{2}\;\int \;\psi \left(r_{pe}\right)\psi \left(r_{p1}\right)B\left(k_{F}\;r_{e1}\right)\;\;d^{3}r_{pe}\;d^{3}r_{p1}\;=\;\frac{2}{\pi }\left[\arctan\left(\nu \right)-\frac{\nu -\frac{8}{3}\nu^{3}-\nu^{5}}{{\left(1+\nu^{2}\right)}^{3}}\right]\;, \end{array}$$

where $\nu =2k_{F}a_{0}={\left(3\pi \rho_{e}/k_{0}\right)}^{1/3}$. In Figure 1, we plot these quantities as a function of the normalized electron density $\rho_{e}/k_{0}$ felt by Ps in the interacting region; both these functions increase with increasing density values, while they vanish at the low-density limit.

Next, let’s calculate the ratio:

(14) $$\frac{\lambda_{\mathrm{ex}}^{3\gamma }}{\lambda_{\mathrm{sym}}}\;=\;-\frac{\lambda_{3\gamma }}{\bar{\lambda }}\;\;\frac{A(\nu )}{\rho_{e}/k_{0}}\;,$$

and similarly for $\lambda_{\mathrm{ex}}^{2\gamma }$. Finally, by inserting these expressions in the left-hand side of Equation (6), we obtain the noteworthy simple formula:

(15) $$\frac{1-\frac{\lambda_{2\gamma }}{\bar{\lambda }}\;\;\frac{A(\nu )}{\rho_{e}/k_{0}}}{1-\frac{\lambda_{3\gamma }}{\bar{\lambda }}\;\;\frac{A(\nu )}{\rho_{e}/k_{0}}}\;=\;K_{M}\left(\lambda_{s},\lambda_{t}\right)\;.$$

In this expression, the dependence on the $P_{\mathrm{out}}$, the probability of finding the Ps center of mass in the interaction region, completely disappears. Hence, for the purpose of using this formula for connecting theoretical findings and experimental data, there is no necessity of making any assumption or hypothesis on the form of the confining potential or on the geometrical shape of the confining nanocavity (note that until now, there was no need to assume a spherical geometry).

**Figure 2:**
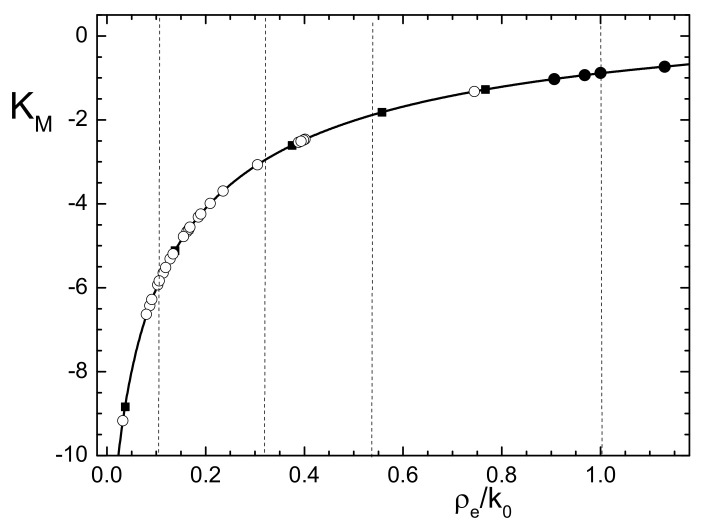
Plot of quantity $K_{M}$ as a function of normalized electron density $\rho_{e}/k_{0}$ from Equation (16). Black circles and squares indicate values corresponding to materials listed in Table 1. Open circles indicate values corresponding to all the materials listed in Table 2. These data are discussed in text. Vertical dashed lines refer to selected values for curves with constant $\rho_{e}/k_{0}$ plotted in Figure 3.

Moreover, this expression connects directly the normalized electron density felt by the Ps with the experimental findings and known quantities, and this can be of paramount importance in applications. A simpler and more useful version of this formula can be derived as follows: by remembering that $\lambda_{2\gamma }\gg \lambda_{3\gamma }$ and that $\bar{\lambda }=\lambda_{2\gamma }/4+3\;\lambda_{3\gamma }/4\approx \lambda_{2\gamma }/4$, the relation in Equation (15) can be reduced in:

(16) $$K_{M}\left(\lambda_{s},\lambda_{t}\right)\;\equiv \;\frac{\lambda_{s}-\lambda_{2\gamma }}{\lambda_{t}-\lambda_{3\gamma }}\;=\;1-4\;\frac{A(\nu )}{\rho_{e}/k_{0}}\;,$$

which explicitly shows the relation between the PALS measurable lifetimes $\lambda_{s}$ and $\lambda_{t}$ with the electron density. A plot of the expression on the right-side of this relation, as a function of the normalized electron density, is given in Figure 2. In this plot, we also indicated with small circles the positions corresponding to the selection of materials listed in Table 1 and Table 2, the PALS data, which are analyzed in the next Section.

Another important consequence of Equation (16) is that, while $\lambda_{t}$ and $\lambda_{s}$ singularly depend on geometrical aspects of the confining medium, i.e., cavity size and shape, their function $K_{M}$ does not. This way, the electron density value obtained by inversion of $K_{M}$ is more significative in materials where a vast range of pore sizes exists. This also means that, generally speaking, for a given material with fixed electronic properties, the annihilation rates turn out to be anticorrelated: bigger cavities correspond to lower $\lambda_{t}$ and bigger $\lambda_{s}$, while vice-versa, smaller cavities correspond to bigger $\lambda_{t}$ and lower $\lambda_{s}$, up to the limit where the overlap with outer electron is so strong that $\lambda_{s}$ equals $\lambda_{t}$ and it is not possible to speak of a spin-polarized Ps [14].

## 4. Connection with Experimental Data on Annihilation Rates

Information on the electron density felt by Ps can therefore be obtained directly from PALS data on measurements of annihilation lifetimes, by inverting Equation (16). In particular, we discuss known results for some solid polymers and molecular crystals with nano- or subnano-voids, for which our theory is well-suited. Their PALS spectra are usually decomposed in 3 or 4 lifetime components. In the common interpretation, these materials show a shorter component of the spectra, $\tau_{1}≳0.125\;$ ns, associated to the *p*-Ps annihilation rate $\lambda_{s}=1/\tau_{1}$. An intermediate lifetime $\tau_{2}∼0.3$–0.5 ns is due to direct positron annihilation, and it is of no interest. The longest $\tau_{3}$ and $\tau_{4}$, which are of the order of 1–5 ns for the materials considered here, can be associated to *o*-Ps annihilating via pickoff process, eventually corresponding to nanocavities with different size distribution. We consider the longest lifetime component as the reference annihilation time for the calculation of the *o*-Ps annihilation rate. Of course, this rate is naturally $\lambda_{t}=1/\tau_{3}$ for materials having spectra with only three components. But in some other materials where the fourth component $\tau_{4}$ is present and falls within the expected range of values, it can be considered on the same foot as the previous data, and we define the annihilation rate as $\lambda_{t}=1/\tau_{4}$.

Before discussing the relation between our model and experimental data, a fundamental comment is in order. Despite the above classification of the lifetimes $\tau_{1}$, $\tau_{3}$, and $\tau_{4}$ being widely accepted, the implications on their relative intensities $I_{1}$, $I_{3}$, and $I_{4}$ of the annihilation channels, as observed in PALS spectra, are rarely considered. Indeed, any model describing *p*-Ps and *o*-Ps formation by unpolarized positrons predicts the ratio $I_{1}/I_{3}=1/3$ for spectra with three components, or $I_{1}/\left(I_{3}+I_{4}\right)=1/3$ in the case of spectra with four components. As a matter of fact, only very few spectra maintain this correct ratio between intensities. This condition could be imposed during the spectrum analysis, but it is common practice to ignore it and let all the intensities vary freely during the fitting procedure, thus improving the fit convergence. This is because sometimes nonphysical values for the lifetimes are obtained by imposing constraints on the intensities.

We stress that, without this rule, $\tau_{1}$ cannot in principle be associated to *p*-Ps, and it is predictable that the component of direct positron annihilation can be mixed with the *p*-Ps one, so introducing a bias on the observed shorter component of the spectra. Hence, in some cases, $\tau_{1}$ values turn out to be too big, with the consequence of nonacceptable or contradictory results about $\rho_{e}/k_{0}$ or about the probability $P_{\mathrm{out}}$. This is what happens with a majority of the materials listed in the Tables that we discuss later.

In Table 1 we report known experimental data on $\tau_{1}$ and $\tau_{3}$ (or $\tau_{4}$) for a first series of materials, with the corresponding estimated values for the quantity $K_{M}$ and the electron density felt by Ps normalized with respect to the vacuum contact density. The first four entries in this Table, designed by black circles in figures, correspond to the only samples in which the intensity rule was imposed; therefore, in these cases, $\tau_{1}$ can be effectively associated to the *p*-Ps lifetime.

In Table 2, we report known experimental data on $\tau_{1}$ and $\tau_{3}$ of some other materials, which are organized in series that underwent different physical or chemical treatments, to study any modifications of the nanovoid structure. In particular, the DOP series corresponds to amorphous polycarbonate polymeric films (CR-39) samples exposed with gamma radiation [31]; the ZMS-5 series corresponds to calcinated zeolites [32]; the series signed Ag/polymer is Ag nanoparticles embedded in a polymer matrix [33]; the series β-As${}_{4}$S${}_{4}$ is mechanochemical milled nanoarsenical pellets [34]; finally the last series corresponds to dimethacrylate composites after photopolymerization [35].

For all these materials we have represented in Figure 2, the found normalized electron density felt by Ps with the corresponding value of the quantity $K_{M}$. In particular, black circles and squares correspond to the first four entries and to the other entries in Table 1, respectively. Open circles correspond to the materials listed in Table 2. The density covers a wide range of values, and the samples respecting the intensity rule (black circles) are placed around $\rho_{e}/k_{0}∼1$ ($\rho_{e}∼270\;\;{\mathrm{nm}}^{-3}$). On the other hand, the majority of the samples listed in Table 2 present a lower density, around $\rho_{e}/k_{0}∼0.2$ ($\rho_{e}∼54\;\;{\mathrm{nm}}^{-3}$), and this can be a result linked to the nonperfect correspondence between $\tau_{1}$ and *p*-Ps lifetime, as discussed above.

**Figure 3:**
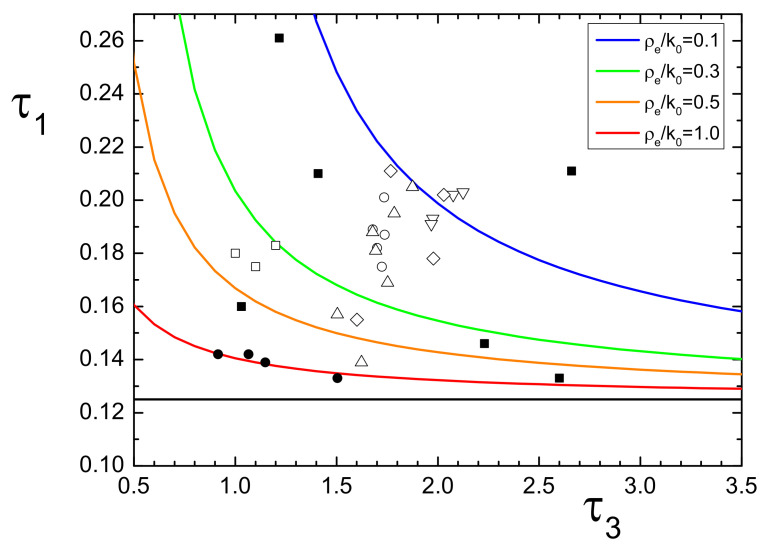
Relationship between $\tau_{1}$ and $\tau_{3}$ lifetimes for some values of $\rho_{e}/k_{0}$ (each represented by a different color). Known experimental data of materials listed in Table 1 are represented by black circles, for data respecting the intensity rule, and by black squares for the other data. Known experimental data of the series of materials listed in Table 2 are represented by open symbols, as in table. Straight black line represents the *p*-Ps lifetime in vacuum (0.125 ns).

Another useful representation of the relation between the experimental lifetime data and the electron density predicted by our model is illustrated in Figure 3. In this figure, the curves represent *p*-Ps and *o*-Ps lifetime components for different fixed values of $\rho_{e}/k_{0}$. In the figure, we also plot points representative of the materials listed in Tables. Regarding the Table 1, while the materials for which the intensity rule was imposed are all placed near the red curve ($\rho_{e}/k_{0}∼1$), the other materials are spread around. On the other hand, regarding the materials in Table 2, this plot shows the modification in the electron density, which can be associated to the diverse treatments on the different family of samples. For example, the DOP series (open circles) shows a slight increase in the density with the radiation exposure; the zeolite ZMS-5 series (open squares), excluding the fourth element of the series, seems to have undergone no changes; the Ag/polymer series (triangle) shows a density much lower with respect to the pure polymer, but increases with the embedded nanoparticle size.

## 5. Spherical Approximation and Determination of Nanocavity Average Sizes

Our model, describing the Ps properties in nanovoids, hence giving information on the electron density surrounding the confined Ps, can also be used to determine nanocavity average sizes. Adopting for definiteness a spherical approximation, and keeping in mind the prescriptions of the TE model, the small interaction region Ω outside a spherical confining cavity of radius $R_{c}$ can be taken as a shell of thickness Δ, and the probability of finding the Ps center of mass in Ω defined in Equation (9) becomes:

(17) $$P_{\mathrm{out}}\;=\;\int_{\Omega }\;{\left|\Psi \left(R\right)\right|}^{2}\;\;d^{3}R\;=\;1-\frac{R_{c}}{R_{c}+\Delta }+\frac{1}{2\pi }\sin\frac{2\pi R_{c}}{R_{c}+\Delta }\;,$$

which is of course the same result of the TE model (see Equation (2) [3,4,18,36]. Note that in that model, it is commonly accepted that Δ=0.166 nm, though this value is well-confirmed only in zeolite samples. Anyway, in absence of other information, we will adopt this value for Δ. Note also that higher values of $P_{\mathrm{out}}$ imply lower values of $R_{c}$ and vice-versa, and in particular, with $P_{\mathrm{out}}=1$, one has $R_{c}=0$ nm.

On the other side, with the help of the approximate formulas for the annihilation contributions $\lambda_{\mathrm{sym}}$, $\lambda_{\mathrm{ex}}^{3\gamma }$, $\lambda_{\mathrm{ex}}^{2\gamma }$, and *S*, expressed by using the auxiliary functions A(ν) and C(ν) defined in the previous section, the *o*-Ps and *p*-Ps total annihilation rates assume the explicit form:

(18) $$\begin{array}{cc} \lambda_{t} & \;=\;\left[1-\frac{P_{\mathrm{out}}\;A\left(\nu \right)}{1-P_{\mathrm{out}}\;C\left(\nu \right)}\right]\lambda_{3\gamma }+\left[\frac{\rho_{e}}{k_{0}}\;\frac{P_{\mathrm{out}}}{1-P_{\mathrm{out}}\;C\left(\nu \right)}\right]\bar{\lambda }\;,\\ \lambda_{s} & \;=\;\left[1-\frac{P_{\mathrm{out}}\;A\left(\nu \right)}{1-P_{\mathrm{out}}\;C\left(\nu \right)}\right]\lambda_{2\gamma }+\left[\frac{\rho_{e}}{k_{0}}\frac{P_{\mathrm{out}}}{1-P_{\mathrm{out}}\;C\left(\nu \right)}\right]\bar{\lambda }\;, \end{array}$$

The main advantage of this formulation is that geometrical effects are well-separated from those due to electron exchange. In fact, geometrical parameters are contained only in the definition of the quantity $P_{\mathrm{out}}$, which depends, in this approximation, on $R_{c}$ and Δ, while the effects of the exchange interaction are represented by the normalized electron density $\rho_{e}/k_{0}$ and by the functions A(ν) and C(ν) which depend on it.

Now, with a little algebra, it is easy to derive $P_{\mathrm{out}}$ as a function of the known electron density $\rho_{e}/k_{0}$ for the materials listed in the two tables by using the experimental data for the annihilation rates. The result is reported in the sixth column of the tables. For the majority of the material samples, the probability $P_{\mathrm{out}}$ comes out well-below 1, as can be expected, and in particular, this happens for the samples obeying the intensity rule, supporting the validity of our theoretical elaboration. But for some other materials, the probability $P_{\mathrm{out}}$ turns out to be very close to 1, and in particular, for the “PPA” in Table 1, the “ZMS5 calc. 6 h”, and the “REHE 600” in Table 2, $P_{\mathrm{out}}$ results greater or equal to 1, a result that is obviously not acceptable. In all these cases, $\tau_{1}$ is greater than 0.2 ns, and hence, probably mixed with another component of the annihilation spectra, as discussed in the Section 3. Therefore, these lifetimes cannot be attributed with certainty to the *p*-Ps lifetime, and our model turns out to give these strange and absurd results in this situation.

By inverting Equation (17) we obtain the data on the nanocavity size $R_{c}$ reported in the seventh column of the tables. Evidently, given that the Ps radius amounts to 0.106 nm, the range of values of $R_{c}$ is between a maximum of near three times Ps radius and a minimum that is slightly less than one half of Ps radius. The values of $R_{c}$ extracted for materials in which the intensity rule was not respected must be considered doubtfully, especially when very small values are obtained, for the reason discussed earlier. On the other hand, the $R_{c}$ values obtained for the four material samples respecting the rule must be considered fully realistic.

Importantly, in literature, the simple TE prescription of Equation (2) is usually used for an estimation of $R_{c}$; hence, considering only the data on *o*-Ps annihilation lifetime. In fact, this is what was done by the authors of some of the papers cited in Table 2, who obtained values greater than ours in almost all cases. However, the simple TE prescription rests on the assumption of a fixed shell thickness Δ=0.166 nm that cannot be fully justified for all the different materials. In fact, the above choice for Δ was calibrated only with zeolites. Therefore, if we consider only these materials, in Table 2 we have three entries identified by the acronyms: “ZMS5 as prep.”, “ZMS5 calc. 2 h”, and “ZMS5 calc. 4 h”. To these samples, in Ref. [32] the authors attribute the following values for the cavity size: 0.143, 0.131, and 0.118 nm, respectively. On the other hand, our estimation gives 0.13, 0.15, and 0.14 nm, respectively. Hence, our results are very similar to those found in the safe range of validity of the simple TE model, confirming the reliability of our treatment. Although the determination of the cavity sizes still requires currently a guess on the shell thickness Δ, as in TE model, we are confident that our model can give correct estimations for $R_{c}$, as well as for the electron density surrounding trapped Ps, once the issues about the significance of $\tau_{1}$ are resolved.

**Figure 4:**
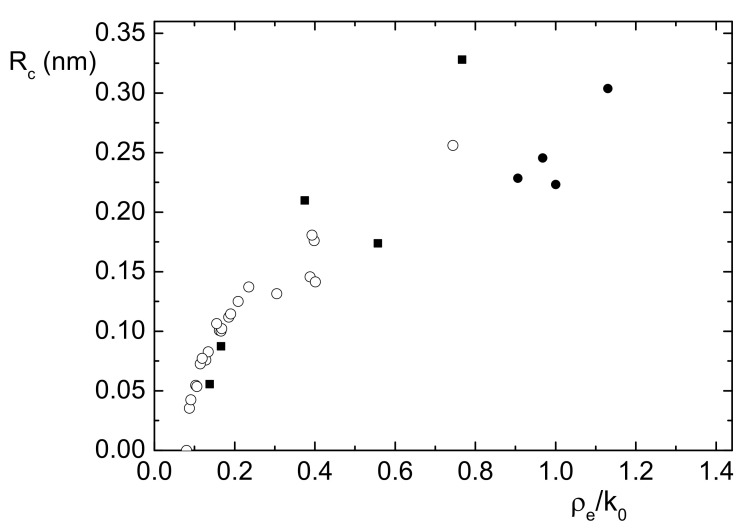
Plot of data on cavity radius $R_{c}$ versus the corresponding data on normalized electron density $\rho_{e}/k_{0}$. For sample materials considered in Table 1, black circles are used for samples respecting the intensity rule, and black squares for others. Sample materials in Table 2 are indicated by open circles.

Finally, it is interesting to study the correlation between the cavity size $R_{c}$ and $\rho_{e}/k_{0}$; this is represented in Figure 4. Apart from the question on the validity of these results, it is apparent that the majority of the materials in Table 2, which have lower values of $\rho_{e}/k_{0}$, show lower values for $R_{c}$, while the materials in Table 1, which respect the intensity rule, stand on an average value around 0.25 nm. This is surprising because one may naively expect that the interaction between Ps and the surroundings may imply that higher electron density would correspond to lower $R_{c}$ and vice-versa.

But, our theory demonstrates that the basic interaction is the exchange correlation between the Ps-electron with electrons outside the subnano pores or confining cavites. Evidently, the contributes to the annihilation rates, $\lambda_{\mathrm{sym}}$, $\lambda_{\mathrm{ex}}^{3\gamma }$, and $\lambda_{\mathrm{ex}}^{2\gamma }$, are explicitly given by Equations (10) and (11), which are roughly proportional to the product of the normalized electron density with the probability $P_{\mathrm{out}}$. Hence these two quantities tend to be anticorrelated, and as a consequence a positive correlation between $\rho_{e}/k_{0}$ and $R_{c}$ is naturally introduced.

A guess about a physical interpretation of these results can be given under the form of these statements. On one hand, for very small pores, if the Ps has to be formed inside them, it means that the surrounding electron density must be low, otherwise the annihilation rates $\lambda_{s}$ and $\lambda_{t}$ tend to become indistinguishable and it is not possible to speak of a confined Ps, as discussed in the previous Section. On the other hand, for larger pores, the Ps must see a greater electron density in the surrounding for observing the modification on the *p*-Ps and *o*-Ps lifetimes with respect to the vacuum values. Of course, this reading of the results can be purely indicative, being subject to the validity of the interpretation of $\tau_{1}$ as the *p*-Ps lifetime.

## 6. Conclusions

We presented a theoretical description of the annihilation behavior of Ps atoms trapped in nano- or subnano-porous materials. Our modelization is based on the unavoidable presence of exchange effects between the Ps-electron and the external electrons in the surface region of the confined nanocavities. This exchange interaction can affect, to some relevant extent, the pickoff annihilation or, more generally, it can affect both the *p*-Ps$\lambda_{s}$ and the *o*-Ps$\lambda_{t}$ annihilation rates, as demonstrated by our theory.

Starting from the explicit formal expressions of the annihilation rates $\lambda_{s}$ and $\lambda_{t}$ found in a previous paper [14], we showed that a simple relation exists between *p*-Ps and *o*-Ps lifetime components in matter and the external electronic density felt by Ps in its position. In fact, this relationship results in being independent from the geometrical properties of the confining medium, and it can be used to gather important information on electron density and on the average cavity radius straight from an (intensity-constrained) experimental PALS spectrum. These results were widely discussed by analyzing some annihilation lifetimes data obtained from known examples of materials like insulators and molecular solids.

As we have pointed out during results analysis, the need for lifetimes components whose intensities satisfy the well-known 1/3 ratio between the *p*-Ps and *o*-Ps observed intensities is of mandatory importance to our model, since without that constraint, any association between observed lifetimes with *p*-Ps or *o*-Ps Ps cannot be fully justified.

Furthermore, despite being a first-order exchange correction, our relationship highlights the fact that the two annihilation channels are deeply interconnected in a way that goes beyond the intensity ratio. In previous literature, this connection was disregarded, and for both channels, intensities and annihilation rates were considered independent. Our framework suggests that more care in fitting PALS spectra should be taken in future experiments to further improve the accuracy and the quantity of extracted information.

## Figures and Tables

**Table 1:** Experimental PALS data for some solid materials. Cases with multiple long lifetime components ($\tau_{3}$ and $\tau_{4}$), for which only long component is considered, are indicated. The first four rows correspond to only samples for which the correct ratio between signal intensities was respected. Fourth and fifth columns report calculated $K_{M}$ and $\rho_{e}/k_{0}$. Sixth and seventh columns are discussed in next section. These materials are represented in Figure 2 and Figure 3, with symbols listed in eighth column of table. Acronyms PMMA, PPD, and PPA stand for Polymethylmethacrylate, 2,5-Diphenyl 1,3,4 oxadiazole, and Atactic polypropylene, respectively.

Name	$\tau_{1}$ (ns)	$\tau_{3,4}$ (ns)	$K_{M}$	$\rho_{e}/k_{0}$	Pout	$R_{c}$ (nm)	Symbol	Ref.
Acenaphtene	0.142	0.915	−0.88	1.00	0.36	0.22	●	[26]
Byphenil	0.139	1.148	−0.93	0.968	0.31	0.25		[26]
Octadecane (solid)	0.133	1.504	−0.73	1.13	0.23	0.30		[27]
2,5-Diphenyloxazole (PPO)	0.142	1.065	−1.03	0.906	0.34	0.23		[28]
PMMA ($\tau_{4}$)	0.146	2.23	−2.61	0.375	0.38	0.21	■	[29]
Polyethylene (PE) ($\tau_{4}$)	0.133	2.60	−1.27	0.767	0.20	0.33		[29]
Naphthalene	0.160	1.030	−1.82	0.557	0.48	0.17		[30]
Butyl-PBD	0.21	1.409	−4.61	0.166	0.79	0.09		[28]
PPD	0.261	1.217	−5.12	0.138	0.91	0.06		[28]
PPA ($\tau_{4}$)	0.211	2.66	−8.84	0.037	1.50	-		[29]

**Table 2:** Experimental PALS data for some solid materials organized in series having undergone different treatments (see text). Fourth and fifth columns report calculated $K_{M}$ and $\rho_{e}/k_{0}$. Sixth and seventh columns are discussed in next section. These series of materials are represented in Figure 3, with the symbols listed in eighth column of the table.

Name	$\tau_{1}$ (ns)	$\tau_{3,4}$ (ns)	$K_{M}$	$\rho_{e}/k_{0}$	Pout	$R_{c}$ (nm)	Symbol	Ref.
DOP-0	0.201	1.735	−5.31	0.128	0.83	0.08	◯	[31]
DOP-250	0.182	1.700	−4.31	0.185	0.69	0.11		
DOP-500	0.187	1.737	−4.66	0.162	0.73	0.10		
DOP-750	0.189	1.679	−4.60	0.166	0.73	0.10		
DOP-1000	0.175	1.724	−3.99	0.209	0.64	0.13		
ZMS5 as prep.	0.183	1.2	−3.07	0.305	0.61	0.13	□	[32]
ZMS5 calc. 2 h	0.175	1.1	−2.53	0.388	0.56	0.15		
ZMS5 calc. 4 h	0.180	1.0	−2.46	0.401	0.58	0.14		
ZMS5 calc. 6 h	0.280	2.04	−9.17	0.032	1.77	−		
pure polymer	0.139	1.623	−1.32	0.744	0.29	0.26	△	[33]
16.4 nm Ag/polymer	0.205	1.875	−5.93	0.103	0.91	0.06		
19.5 nm Ag/polymer	0.195	1.785	−5.19	0.134	0.80	0.08		
22.0 nm Ag/polymer	0.181	1.694	−4.24	0.190	0.68	0.11		
25.8 nm Ag/polymer	0.188	1.679	−4.55	0.168	0.73	0.10		
29.7 nm Ag/polymer	0.157	1.504	−2.48	0.398	0.47	0.18		
33.3 nm Ag/polymer	0.169	1.753	−3.70	0.235	0.59	0.14		
β-As${}_{4}$S${}_{4}$ REHE−0	0.193	1.976	−5.65	0.114	0.84	0.07	▽	[34]
β-As${}_{4}$S${}_{4}$ REHE−200	0.191	1.968	−5.52	0.119	0.83	0.08		
β-As${}_{4}$S${}_{4}$ REHE−500	0.202	2.076	−6.42	0.087	0.97	0.04		
β-As${}_{4}$S${}_{4}$ REHE−600	0.203	2.125	−6.63	0.080	1.00	0.		
Dipol-0	0.178	1.978	−4.78	0.155	0.71	0.11	♢	[35]
Dipol-60	0.155	1.601	−2.51	0.393	0.46	0.18		
eCTA-0	0.202	2.030	−6.28	0.091	0.95	0.04		
eCTA-60	0.211	1.768	−5.84	0.106	0.27	0.05		

## Appendix A. Calculation of the Exchange Contribution to Annihilation Rates

The treatment of the antisymmetry problem in many-body physics is essentially based on the methods of the symmetry-adapted perturbation theory (SAPT) [15] An accurate review of SAPT theories is beyond the scope of the present discussion and can be found in Ref. [37]. In all SAPT formulations, the first-order correction to the expected values of an electron composite system, due to an interaction operator V, reads:

(A1) $$E^{\left(1\right)}\;=\;\frac{\langle \psi^{\left(0\right)}|V|A\psi^{\left(0\right)}\rangle }{\langle \psi^{\left(0\right)}|A\psi^{\left(0\right)}\rangle }\;.$$

where $\psi^{\left(0\right)}$ represents a suitable unperturbed wavefunction, and the operator A is an intermolecular antisymmetrizer operator, defined as

(A2) $$A=\frac{1}{N!}\sum_{p}{\left(-1\right)}^{p}P\;,$$

with *P* representing a permutation operator of *N* electrons, while ${\left(-1\right)}^{p}$ stands for the parity of the permutation. Note that with this definition, A is idempotent, i.e., $A^{2}=A$. The normalization is guaranteed by the factor $\langle \psi^{\left(0\right)}|A\psi^{\left(0\right)}\rangle =\langle \psi^{\left(0\right)}A|A\psi^{\left(0\right)}\rangle$ at the denominator of Equation (A1) (which is called an intermediate–normalization condition in literature [37]).

As an unperturbed wavefunction of the system, we consider the product of a Ps wavefunction $\Psi_{jm}$ times the (antisymmetric) ground state ϕ of the *N*-electron system:

(A3) $$\psi_{jm}^{\left(0\right)}\left(p,e,1,\cdots ,N\right)=\Psi_{jm}\left(p,e\right)\phi \left(1,2,\cdots ,N\right)\;,$$

where j,m are the Ps spin *S* and spin projection $S_{z}$ quantum numbers (j=1 for *o*-Ps and j=0 for *p*-Ps), and we use, from now on, the compact notation $p=(r_{p},\sigma_{p})$ and $e=(r_{e},\sigma_{e})$ for the spin-spatial coordinates of the Ps positron and electron, respectively, while numbers refer to the other electrons. This wavefunction is by construction antisymmetric with respect to the exchange of any two electrons in 1,…,N since:

(A4) ϕ(1⋯i⋯j⋯N)=−ϕ(1⋯j⋯i⋯N)∀i,j,

but it is not antisymmetric with respect to the exchange with Ps electron.

It is important to introduce some descriptive quantities of the many body outer electron system. The external electron bulk density is connected to the square modulus of the *N*-electron normalized wavefunction and is commonly defined in the density functional theory (DFT) as:

(A5) $$n\left(r\right)=N\;\sum_{\sigma_{1}}\int {\left|\phi \left(r,\sigma_{1},2,\cdots ,N\right)\right|}^{2}\;d2\ldots \;dN\;.$$

Here and in the following, we use the compact notation $\int \;di=\sum_{\sigma_{i}}\int \;d^{3}r_{i}$ to represent both spin summation and spatial integration. Other useful quantities are the *reduced density matrices* (RDM), which offers a convenient way of describing the internal structure of a many body system of *N* indistinguishable particles without the complete knowledge of its wavefunction. The simplest RDM is the one-body reduced density matrix (1RDM), which is defined as:

(A6) $$\Gamma^{\left(1\right)}\left(x;y\right)\;=\;N\;\int \phi \left(x,2,\cdots ,N\right)\;\phi^{*}\left(y,2,\cdots ,N\right)\;\;d2\cdots \;dN\;,$$

where *x* and *y* denote the couple $\left(r_{x},\sigma_{x}\right)$ and $\left(r_{y},\sigma_{y}\right)$. The 1RDM is composed in principle by the 4 components $\Gamma_{↑↑}^{\left(1\right)}$, $\Gamma_{↑↓}^{\left(1\right)}$, $\Gamma_{↓↑}^{\left(1\right)}$ and $\Gamma_{↓↓}^{\left(1\right)}$ resulting from expansion in a complete set of spin functions:

(A7) $$\Gamma^{\left(1\right)}\left(x;y\right)=\sum_{ij}\Gamma_{ij}^{\left(1\right)}\left(r_{x};r_{y}\right)\;s_{i}\left(\sigma_{x}\right)\;s_{j}\left(\sigma_{y}\right)\;,$$

where *i* and *j* may represent ↑ or ↓ spin states. Furthermore, we can define the spatial 1RDM by summing $\Gamma^{\left(1\right)}$ over the spin variables:

(A8) $$\Gamma^{\left(1\right)}\left(r_{x};r_{y}\right)\;=\;\sum_{\sigma_{x},\sigma_{y}}\;\Gamma^{\left(1\right)}\left(x;y\right)\;\equiv \;\Gamma_{↑↑}^{\left(1\right)}\left(r_{x};r_{y}\right)+\Gamma_{↓↓}^{\left(1\right)}\left(r_{x};r_{y}\right)\;,$$

where the last passage is valid if no spin mixing potential appears in the hamiltonian of the bulk system, as assumed here. In this case, the wavefunction ϕ is an eigenstate of $S_{z}$ and the two spin channels decouple, so that $\Gamma_{↑↓}^{\left(1\right)}=\Gamma_{↓↑}^{\left(1\right)}=0$ [38]. The diagonal part of the spatial 1RDM is just the electron density defined in Equation (A5):

(A9) $$n\left(r\right)=\Gamma^{\left(1\right)}\left(r;r\right)=n_{↑}\left(r\right)+n_{↓}\left(r\right)\;,$$

where $n_{↑}$($n_{↓}$) is the local spin up(down) density.

To calculate the exchange correlation contributions to the annihilation rates, we apply the SAPT to the annihilation operator given by [16]:

(A10) $${\hat{\lambda }}_{i}=8\pi a_{0}^{3}\;\;\delta^{3}\left(r_{p}-r_{i}\right)\;\left[\frac{1-\Sigma_{p,i}}{2}\lambda_{2\gamma }+\frac{1+\Sigma_{p,i}}{2}\lambda_{3\gamma }\right]\;,$$

where $8\pi a_{0}^{3}$ is the inverse contact density of unperturbed positronium, $r_{p}$ and $r_{i}$ are positron and electrons coordinates, respectively, and $\Sigma_{p,i}$ is the spin exchange operator. Here, $\hat{\lambda }$ is basically a “contact operator”, being a linear combination of delta functions of the electron-positron distance. The spin exchange operator Σ guarantees that the antisymmetric spin configuration annihilates via 2γ emission, while the symmetric spin configuration via 3γ emission. It is easy to see that this form of ${\hat{\lambda }}_{i}$ gives the correct annihilation rates for *p*-Ps and *o*-Ps states in vacuum.

After separating spatial and spin part of the wavefunction, for example $\Psi_{jm}\left(p,e\right)=\Psi \left(r_{p},r_{e}\right)\chi_{jm}\left(\sigma_{p},\sigma_{e}\right)$, using the above definitions and assuming uniform spin distributions of external electrons density:

(A11) $$n_{↑}\left(r\right)\;=\;n_{↓}\left(r\right)\;=\;\frac{1}{2}n\left(r\right)\;,$$

we obtain that the overall correction to the annihilation rate can be expressed as the sum of three different contributions [14]:

(A12) $$\lambda^{\left(1\right)}=\frac{\lambda_{\mathrm{sym}}+\lambda_{\mathrm{ex}}+\lambda_{\mathrm{ex}-\mathrm{po}}}{1-S}\;,$$

where *S* is the overlap parameter, which comes from the normalization factor:

(A13) $$S\;=\;\int \Psi_{jm}^{*}\left(p,e\right)\Psi_{jm}\left(p,1\right)\;\Gamma^{\left(1\right)}\left(e;1\right)\;dp\;de\;d1\;.$$

This parameter becomes important when Ps approaches the external electronic system, where its wavefunction will begin to “overlap” with the system’s one and exchange correlation effects must be considered.

The first term of the sum in Equation (A12) is the symmetric contribution:

(A14) $$\begin{array}{cc} \lambda_{\mathrm{sym}}\;= & \;8\pi a_{0}^{3}\;\int {\left|\Psi \left(r_{p},r_{e}\right)\right|}^{2}\delta \left(r_{1}-r_{p}\right)\left[\lambda_{3\gamma }\;n_{↑}\left(r_{1}\right)+\frac{\lambda_{2\gamma }+\lambda_{3\gamma }}{2}\;n_{↓}\left(r_{1}\right)\right]\;d^{3}r_{p}\;d^{3}r_{e}\;d^{3}r_{1}\\ = & \;8\pi a_{0}^{3}\;\;\bar{\lambda }\;\int {\left|\Psi \left(r_{p},r_{e}\right)\right|}^{2}n\left(r_{p}\right)\;d^{3}r_{p}\;d^{3}r_{e}\;, \end{array}$$

resulting proportional to the averaged annihilation rate $\bar{\lambda }$ defined in Equation (3).

The second term of the sum is the exchange contribution, which can be written in a simple form containing the 1RDM (A6):

(A15) $$\begin{array}{cc} \lambda_{\mathrm{ex}}^{2\gamma }\; & =\;-8\pi a_{0}^{3}\;\;\lambda_{2\gamma }\int \;\Psi^{*}\left(r_{p},r_{e}\right)\Psi \left(r_{p},r_{p}\right)\;\Gamma_{↑↑}^{\left(1\right)}\left(r_{e};r_{p}\right)\;\;d^{3}r_{p}\;d^{3}r_{e}\\ \lambda_{\mathrm{ex}}^{3\gamma }\; & =\;-8\pi a_{0}^{3}\;\;\lambda_{3\gamma }\int \;\Psi^{*}\left(r_{p},r_{e}\right)\Psi \left(r_{p},r_{p}\right)\;\Gamma_{↑↑}^{\left(1\right)}\left(r_{e};r_{p}\right)\;\;d^{3}r_{p}\;d^{3}r_{e}\;, \end{array}$$

for the two cases of *p*-Ps and *o*-Ps, respectively. The evident parallelism in the form of these expression is a consequence of the uniform spin distribution and of the fact that $\Gamma_{↑↑}^{\left(1\right)}=\Gamma_{↓↓}^{\left(1\right)}$.

The exchange-correlation contribution $\lambda_{\mathrm{ex}-\mathrm{po}}$ can be calculated in the same way, resulting in a dependence on a two-body reduced density matrix $\Gamma^{\left(2\right)}$ of the system. As a matter of fact, the result contains spatial integrals over Ps wavefunctions like $\Psi^{*}\left(r_{p},r_{e}\right)\Psi \left(r_{p},r_{2}\right)$ [14] that, by construction, vanish exponentially at large interparticle separation i.e., when $r_{pe},r_{p2}≳2a_{0}$ (the Bohr radius for positronium is twice that of hydrogen). Hence, we can assume that the integration domain is extremely reduced, thus making $\lambda_{\mathrm{ex}-\mathrm{po}}$ a higher order contribution to the annihilation rate, negligible with respect to the other two contributes.

## Appendix B. The Local Density Approximation

To determine the 1RDM one can consider the so–called *local density approximation* (LDA). In LDA, the properties of an electronic system with a density profile n(r) are locally modeled similarly to a free electron gas with the same density. In this simple picture, the 1RDM has an analytical expression [39]:

(A16) $$\Gamma^{\left(1\right)}\left(x;y\right)=\delta_{\sigma_{x}\sigma_{y}}\frac{n(R_{xy})}{2}\;B\left(k_{F}\left(R_{xy}\right)\;\left|r_{xy}\right|\right)\;,$$

where $k_{F}\left(R_{xy}\right)={\left(3\pi^{2}n\left(R_{xy}\right)\right)}^{1/3}$ is a “local” Fermi momentum and

$$B\left(x\right)=3\frac{\sin(x)-x\cos(x)}{x^{3}}\;.$$

The notation of the spatial coordinates is as follows:

$$R_{xy}\;=\;\frac{x+y}{2}\;;\;r_{xy}\;=\;x-y\;,$$

to denote the average and the relative position of two particles *x* and *y*, respectively. Within the usual assumption of uniform spin distribution, the spatial 1RDM is:

$$\Gamma^{\left(1\right)}\left(R_{xy};r_{xy}\right)=\Gamma_{↑↑}^{\left(1\right)}\left(R_{xy};r_{xy}\right)+\Gamma_{↓↓}^{\left(1\right)}\left(R_{xy};r_{xy}\right)\;.$$
